# Impaired complement regulation drives chronic lung allograft dysfunction after lung transplantation

**DOI:** 10.1172/JCI188891

**Published:** 2025-11-11

**Authors:** Hrishikesh S. Kulkarni, Laneshia K. Tague, Daniel R. Calabrese, Fuyi Liao, Zhiyi Liu, Lorena Garnica, Nishanth R. Shankar, Xiaobo Wu, Devesha H. Kulkarni, Aayusha Thapa, Dequan Zhou, Yan Tao, Victoria E. Davis, Cory T. Bernardt, Derek E. Byers, Catherine Chen, Howard J. Huang, Chad A. Witt, Ramsey R. Hachem, Daniel Kreisel, John P. Atkinson, John R. Greenland, Andrew E. Gelman

**Affiliations:** 1 Department of Medicine, Washington University School of Medicine, St. Louis, Missouri, USA.; 2 Department of Medicine, David Geffen School of Medicine at UCLA, Los Angeles, CA, USA.; 3 Department of Medicine, UCSF, San Francisco, California, USA.; 4 Medical Service, Veterans Affairs Health Care System, San Francisco, California, USA.; 5 Department of Surgery and; 6 Department of Pathology & Immunology, Washington University School of Medicine, St. Louis, Missouri, USA.; 7 Department of Internal Medicine, University of Texas Southwestern Medical Center, Dallas, Texas, USA.; 8 Department of Medicine, Houston Methodist Hospital, Houston, Texas, USA.; 9 Department of Internal Medicine, University of Utah Spencer Fox Eccles School of Medicine, Salt Lake City, Utah, USA.

**Keywords:** Immunology, Pulmonology, Complement, Organ transplantation

## Abstract

A greater understanding of chronic lung allograft dysfunction (CLAD) pathobiology, the primary cause of death after lung transplantation (LTx), is needed to improve outcomes. The complement system links innate to adaptive immune responses and is activated early after lung transplantation to form C3 convertase, a critical enzyme that cleaves the central complement component C3. We hypothesized that LTx recipients with a genetic predisposition to enhanced complement activation have worse CLAD-free survival mediated through increased adaptive alloimmunity. We interrogated a known functional C3 polymorphism (C3 R102G) that increases complement activation through impaired C3 convertase inactivation in 2 independent LTx recipient cohorts. C3 R102G, identified in at least 1 of 3 LTx recipients, was associated with worse CLAD-free survival, particularly in the subset of recipients who developed donor-specific antibodies (DSAs). In a mouse orthotopic LTx model, impaired recipient complement regulation led to B cell–dependent CLAD pathology despite moderate differences in graft-infiltrating effector T cells. Dysfunctional complement regulation promoted intragraft accumulation of memory B cells and Ab-secreting cells, leading to increased local and circulating DSA levels in mice. In summary, genetic predisposition to complement activation is associated with an increased humoral response and worse CLAD-free survival.

## Abstract 2

Lung transplant recipients genetically predisposed to impaired complement regulation demonstrate worse chronic rejection-free survival. In an orthotopic mouse lung transplant model, this phenotype is associated with intragraft B cell-activation and donor-specific antibodies.

## Graphical abstract

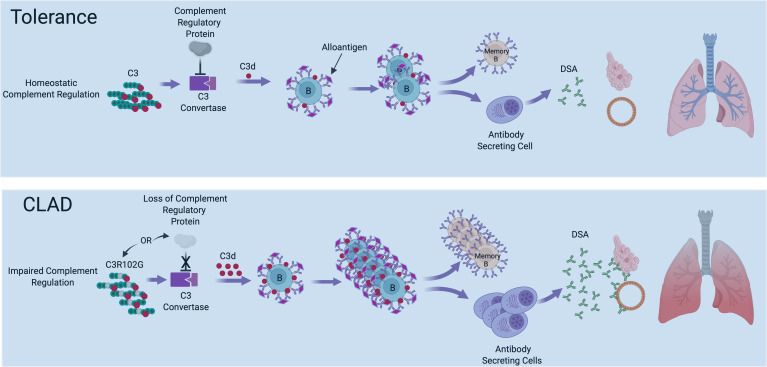

## Introduction

Chronic lung allograft dysfunction (CLAD) is the primary cause of long-term morbidity and mortality after lung transplantation (LTx) (1, 2). CLAD commonly presents with a constrictive bronchiolitis pathology and is associated with poor survival (3). However, it is unclear why some LTx recipients progress to CLAD whereas others do not. CLAD is also a heterogenous syndrome with greater or lesser involvement of donor-specific antibodies (DSAs) and alveolar space, leading to Ab-mediated rejection (AMR) and restrictive allograft syndrome (RAS) (4–6). Crosstalk between innate and adaptive immunity via the complement system may be necessary for driving CLAD (7–9). Although T cells are required for acute and chronic rejection, there is increasing evidence that B cells contribute to poor LTx survival, potentially through AMR (10–12). Additionally, mouse models of LTx have demonstrated B cell–dependent CLAD-like pathology (13–15).

The complement system comprises more than 50 proteins that are activated, amplified, and deposited onto pathogens and dying cells to facilitate their clearance by phagocytosis (16). This system can also be activated in transplantation via Ab-dependent and Ab-independent pathways (17–20). C3a and C5a are cleaved from C3 and C5 upon activating this cascade and can engage with their cognate receptors to influence alloimmune responses (21). Engagement of these receptors also drives profibrotic responses (22, 23). At the same time, the key complement protein C3 is required for the survival of both immune (24) and nonimmune (25–27) cells and modulates effector cell function (28).

A series of both membrane-bound and fluid-phase regulators controls complement activation and facilitates tissue homeostasis (29). Genetic and acquired deficiencies in these regulators increase complement activation, resulting in augmented tissue damage (30, 31). CD46, a membrane regulator, is a cofactor for fluid-phase regulators such as Factor I to cleave C3b and C4b and inactivates C3 convertase to attenuate complement activation. Crry, a murine ortholog of CD46, prevents complement activation on the vascular endothelium (32). Crry is downregulated during lung injury (33) and has also been used to ameliorate lung injury (34, 35).

The role of complement regulation in CLAD remains undefined. Given the centrality of C3 in the cascade, we analyzed a functional polymorphism in C3 (*rs2231099*, C3 R102G) that has a known minor allele frequency of at least 10% in several large cohorts. This polymorphism results in increased complement activation due to the reduced ability of the fluid-phase regulator Factor H to inactivate C3 convertase (36). Previous work has shown that the C3 R102G polymorphism is linked to the development of age-related macular degeneration and other complement-related conditions (37). We hypothesized that C3 R102G is associated with CLAD development. We show that recipients carrying the C3 R102G polymorphism in 2 independent human cohorts had worse CLAD-free survival, and these outcomes were more pronounced in the setting of DSAs. Additionally, using an orthotopic mouse LTx model, we demonstrate that recipients deficient in the complement regulator Crry developed CLAD caused by elevated intragraft B cell activation and high DSA levels (38, 39).

## Results

### A functional polymorphism in the complement component C3 gene is observed in at least 1 in 3 LTx recipients.

**Figure 1:**
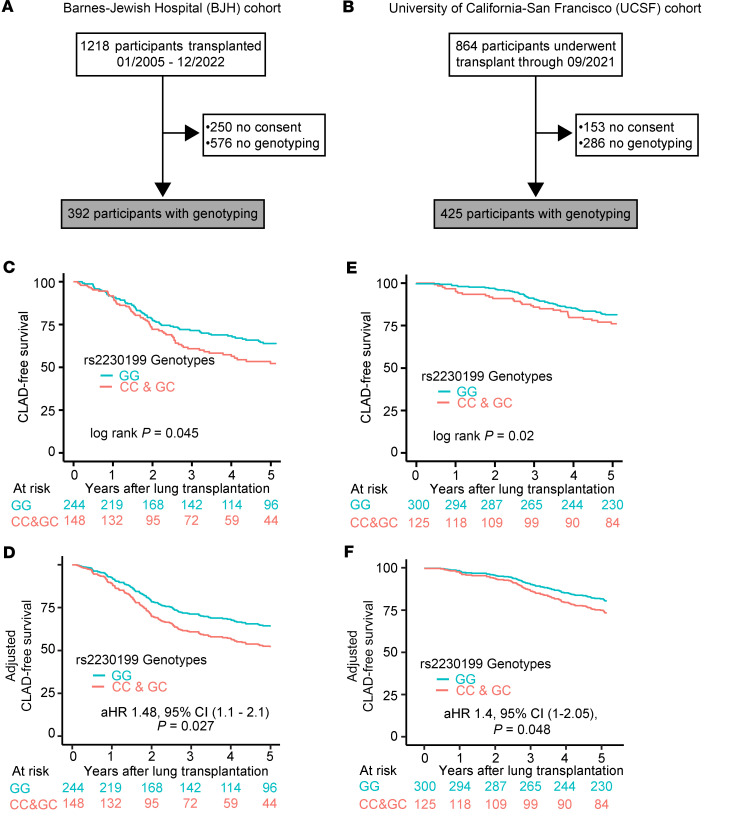
A functional C3 polymorphism confers increased risk of CLAD or death in 2 independent cohorts. Consolidated Standards of Reporting Trials diagrams for the Washington University/BJH (**A**) and UCSF cohorts (**B**). (**C** and **E**) Kaplan-Meier plot of survival from CLAD or death in the BJH (**C**) and UCSF (**E**) cohorts stratified by *rs2230199* genotypes. (**D** and **F**) Kaplan-Meier plot based on the multivariate Cox regression analysis of CLAD-free survival in the BJH (**D**) and UCSF (**F**) cohorts by *rs2230199* status. The *P* value was determined by log-rank test.

Within the LTx cohort at Washington University/Barnes-Jewish Hospital (BJH), we identified 392 participants with available genotyping (Figure 1A). Among these recipients, 244 (62.2%) had wild-type C3 (G/G); thus, the recipient *rs2230199* (G>C) SNP had a minor allele frequency (allele C) of 37.8%. Baseline characteristics for participants stratified by genotype are shown in Table 1 and were similar across both genotypes.

Within the UCSF LTx cohort, we identified 425 participants with available genotyping (Figure 1B). The recipient *rs2230199* (G>C) SNP had a minor allele frequency (allele C) of 29.4%. Baseline characteristics for participants stratified by genotype are shown in Table 2. Notably, we found higher minor allele frequencies among White recipients and lower minor allele frequencies among Black recipients. These distributions are consistent with those reported in aggregated global genomic databases (40). There were no other differences in LTx baseline characteristics across genotypes.

### C3 R102G confers increased risk of CLAD or death in 2 independent cohorts.

We next examined whether the *rs2230199* C3 minor allele, which predisposes to increased complement activation, was associated with decreased CLAD-free survival. In the BJH cohort, the C3 R102G polymorphism was associated with an increased risk for CLAD or death (Figure 1C). In a multivariable Cox-proportional hazards model consisting of C3 *rs2230199* genotype, age, sex, race, transplant diagnosis, DSAs, and Gram-negative infection, C3 R102G remained associated with increased risk of CLAD or death (Figure 1D and Table 3). The presence of post-transplant DSAs (adjusted hazard ratio [aHR] 1.74; 95% CI, 1.24–2.45; *P* = 0.001) or post-transplant Gram-negative infection (aHR 1.44; 95% CI, 1.01–2.06; *P* = 0.044) also was associated with a significantly increased risk of CLAD or death. A sensitivity analysis removing the presence of DSAs among the list of variables demonstrated that C3 R102G was still associated with an increased risk of CLAD or death (aHR 1.52; 95% CI, 1.08–2.15; *P* = 0.016). A similar analysis removing the presence of Gram-negative infection among the list of variables demonstrated that C3 R102G remained associated with an increased risk of CLAD or death (aHR 1.43; 95% CI, 1.02–2.02; *P* = 0.04).

We examined if R102G also was associated with differential CLAD-free survival in the UCSF cohort. Figure 1E shows that participants homozygous for C3 R102G had an increased risk for CLAD or death (HR 1.43; 95% CI, 1–2.05; adjusted *P* = 0.048). This multivariable Cox-proportional hazards model also consisted of C3 *rs2230199* genotype, age, sex, race, transplant diagnosis, DSAs, and Gram-negative infection (Figure 1F and Table 4).

### CLAD-free survival association with recipient C3 R102G is linked to DSAs.

**Figure 2:**
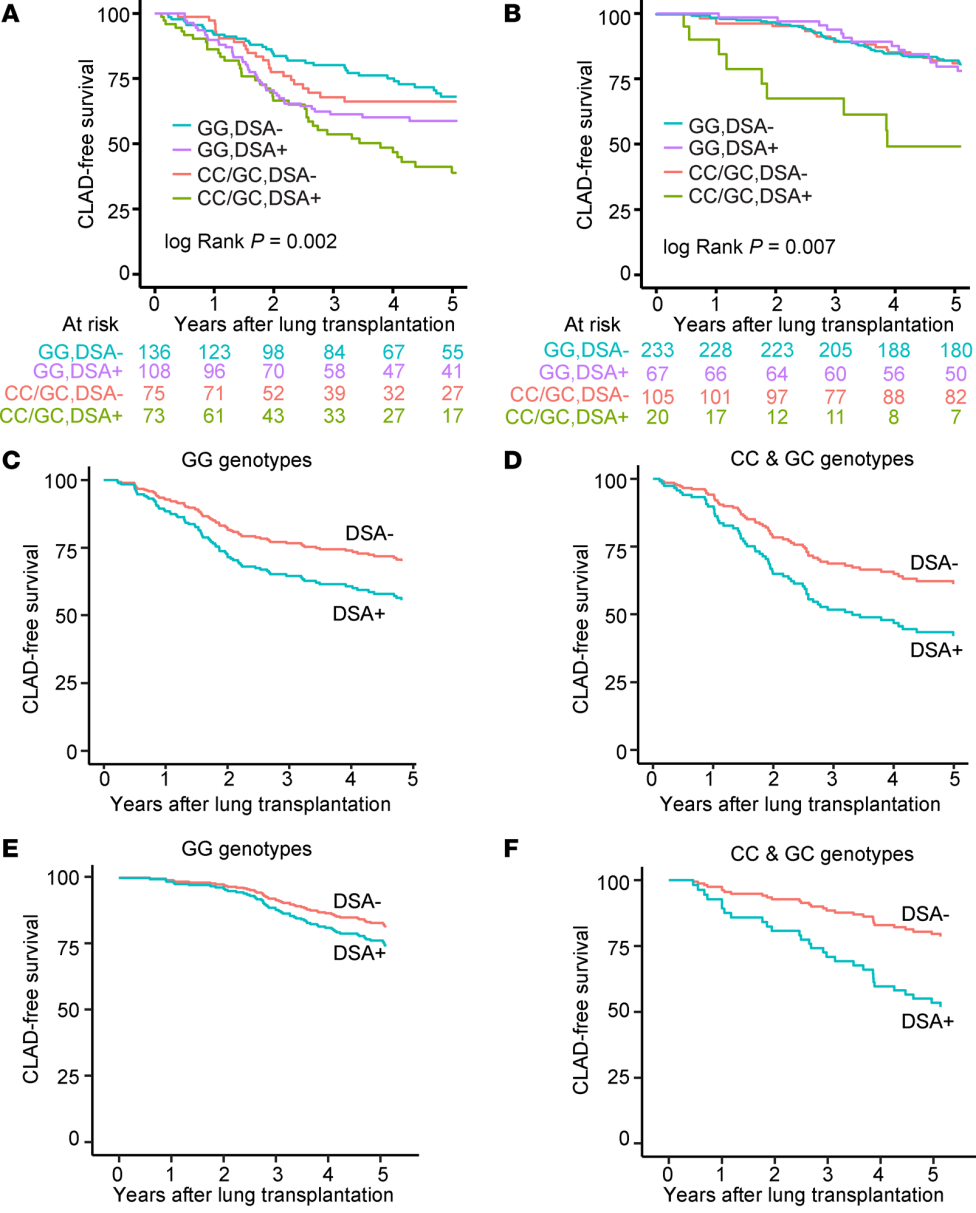
Complement-mediated CLAD or death is dependent on DSAs. CLAD-free survival was worse in the recipients with DSAs and the C3 R102G polymorphism (CC/GC). The BJH (**A**) and UCSF (**B**) cohorts were stratified by DSA-negative and DSA-positive recipients. The *P* value was determined by log-rank test. (**C** and **D**) Kaplan-Meier plot based on the multivariate Cox regression analysis of CLAD-free survival in the UCSF cohort (**C** and **D**) and BJH (**E** and **F**), separated by genotype and stratified by DSA status.

Based on work published by us and others reporting that anti-HLA DSAs are a known risk factor for CLAD (11, 41), we hypothesized that the increased risk of CLAD or death in recipients with C3 R102G would be limited to those with DSAs. In the BJH cohort, 181 LTx recipients (46.2%) had definite DSAs during their follow-up. CLAD-free survival analysis accounting for both DSAs and *rs2230199* genotype demonstrated that recipients with C3 R102G and DSAs had the worst CLAD-free survival (Figure 2A). Among recipients without DSAs, we did not observe a difference in risk for CLAD or death based on the C3 *rs2230199* genotype (HR 1.21; 95% CI, 0.72–2.04; *P* = 0.48). However, among the participants who developed DSAs and had the C3 R102G polymorphism, there was an increased risk for CLAD or death (HR 1.52; 95% CI, 0.99–2.34; *P* = 0.05), with the difference in CLAD-free survival primarily being observed starting at approximately 2.5 years after transplantation.

These observations also held true in the UCSF cohort, wherein 87 LTx recipients (20.5%) had evidence of DSAs at some point during their post-transplant course. Among participants without DSAs there was no different risk for CLAD or death by the *rs2230199* genotype (aHR 1.2; 95% CI, 0.85–1.9; adjusted *P* = 0.24) (Figure 2B). However, among the participants who developed DSAs and had a CC or a GC allele, there was a 3.2-fold increased risk for CLAD or death (aHR 3.2; 95% CI, 1.6–6.2; adjusted *P* = 0.0007). These models were adjusted for age, sex, race, transplant diagnosis, and Gram-negative infection. These findings suggest the impact of the *rs2230199* SNP is dependent upon DSA development.

We next examined CLAD-free survival separately in recipients based on the presence or absence of C3 R102G. Recipients in the UCSF cohort who lacked C3 R102G (i.e., GG genotype) did not demonstrate a significant difference in CLAD-free survival irrespective of their DSA status (Figure 2C). In comparison, recipients with post-transplant DSAs and the C risk allele (i.e., CG or CC genotype) were at a 3-fold higher risk for worse CLAD-free survival after adjusting for age, sex, race, transplant diagnosis, and Gram-negative infection (Figure 2D). The results also held true in the BJH cohort, in which recipients with post-transplant DSAs and the C risk allele had worse CLAD-free survival (aHR 1.9; 95% CI, 1.1–3.3; adjusted *P* = 0.02; Figure 2, E and F). These data suggest the worst long-term outcomes occurred in those who developed post-transplant DSAs and harbored the *rs2230199* SNP.

### Impaired complement regulation promotes CLAD in the mouse orthotopic LTx model.

**Figure 3:**
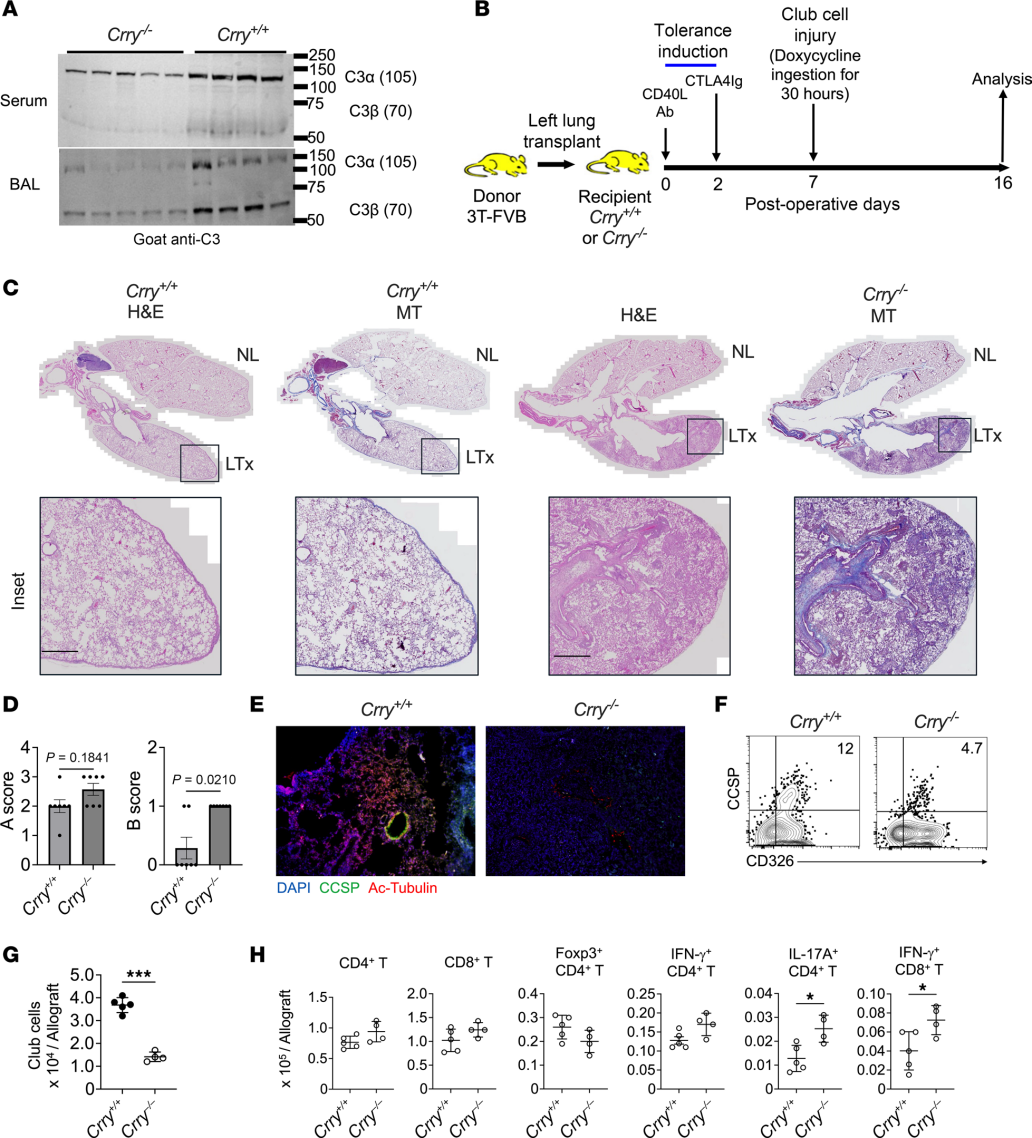
Dysregulated complement activation promotes CLAD in a mouse LTx model. (**A**) Immunoblot of serum and BAL C3-α and -β fragments from resting *Crry*^+/+^ and *Crry^–/–^* mice (*n* = 4/group). The data shown are representative results of 2 experiments. (**B**) Mouse orthotopic left LTx model of CLAD. (**C**) H&E and Masson’s trichrome (MT) staining of a heart-lung block from POD 16 *Crry*^+/+^ and *Crry^–/–^* allograft recipients. Scale bars: 250 μm. The histology shown is a representative result of at least 6 transplants per group. (**D**) POD 16 blinded A (vascular) and B (airway) ISHLT consensus pathological scoring (*n* = 7/group). (**E**) immunofluorescent staining of POD 16 allograft club and ciliated cells using Abs specific for CCSP and acetylated tubulin (Ac-Tubulin). The images shown are representative results of 5 transplants per group. (**F**) A representative flow cytometry plot gating strategy for identifying and (**G**) quantifying POD 16 CD326^+^ CCSP^+^ (club) cells in POD 16 allografts (*n* = 4/group). (**H**) POD 16 intragraft total and indicated subset CD4^+^ and CD8^+^ T cell numbers (*n* = 4/group). Bar graphs and dot plots show mean ± SD for (**D**) Mann-Whitney *U* test and (**G** and **H**) Welch’s *t* test. *P < 0.05, ***P < 0.001. NL, native lung.

The C3 R102G polymorphism lacks a synonymous variant in mice. However, mice deficient in Crry (*Crry^–/–^*) have increased complement activation due to impaired Factor I–mediated cleavage of C3b and C4b, resulting in dysregulated C3 convertase formation on the membrane (38). As a result, these mice have more C3 consumption and lower circulating levels of C3 (39, 42), as well as attenuated levels of C3 in their bronchoalveolar lavage (BAL) (Figure 3A). Given our prior findings that C3 is required for epithelial cell survival (27) and that C3 R102G predisposes to impaired complement regulation (36), we asked if CLAD development is regulated by complement activation. For this purpose, we used a previously established orthotopic mouse LTx model of CLAD (43, 44) (Figure 3B). Wild-type *Crry^+/+^* and *Crry^–/–^* recipients, both on a B6 background (H-2^b^), were engrafted with major-mismatched left lungs encoding 3 transgenes (3T-FVB; H-2^q^): a reverse tetracycline activator gene driven by the club cell secretory protein promoter, a Cre recombinase gene under the control of the reverse tetracycline activator, and a lox-P activated diphtheria toxin A gene. These transgenes induce graft-specific club cell injury and depletion in B6 recipients following 2.5 days of doxycycline (DOX) ingestion, leading to severe CLAD development with a combination of obliterative and restrictive fibrotic pathology that is dependent on alloimmune responses to a major histocompatibility class mismatch. In contrast, 30 hours of DOX ingestion only induces minimal club cell depletion and results in markedly less allograft injury.

After induction of immunosuppression-mediated lung allograft acceptance, recipients ingested DOX on postoperative day (POD) 7 for 30 hours; on POD 16, allografts were examined for histological evidence of CLAD. We observed more evidence of peribronchiolar fibrosis, obliterative airway disease, scarring of the alveolar parenchyma, and collagen deposition in allografts of *Crry^–/–^* when compared with wild-type recipients (Figure 3C and Supplemental Figure 1). Blinded grading for LTx rejection using consensus International Society for Heart and Lung Transplantation (ISHLT) criteria (45) showed significantly higher “B” airway inflammation in allografts after transplantation into *Crry^–/–^* relative to wild-type recipients (Figure 3D). Consistent with these findings, club cells failed to fully reconstitute the graft epithelium in *Crry^–/–^* hosts. Some club cells were observed scattered throughout the interstitium, suggesting epithelial repair is inhibited by augmented complement activation (Figure 3, E–G). Similar to previous findings in humans with CLAD (46, 47), there were more intragraft T_h_17 and IFN-γ^+^ CD8^+^ T cells in mouse recipients lacking Crry (Figure 3H).

### Impaired complement regulation promotes CLAD in a B cell–dependent manner.

**Figure 4:**
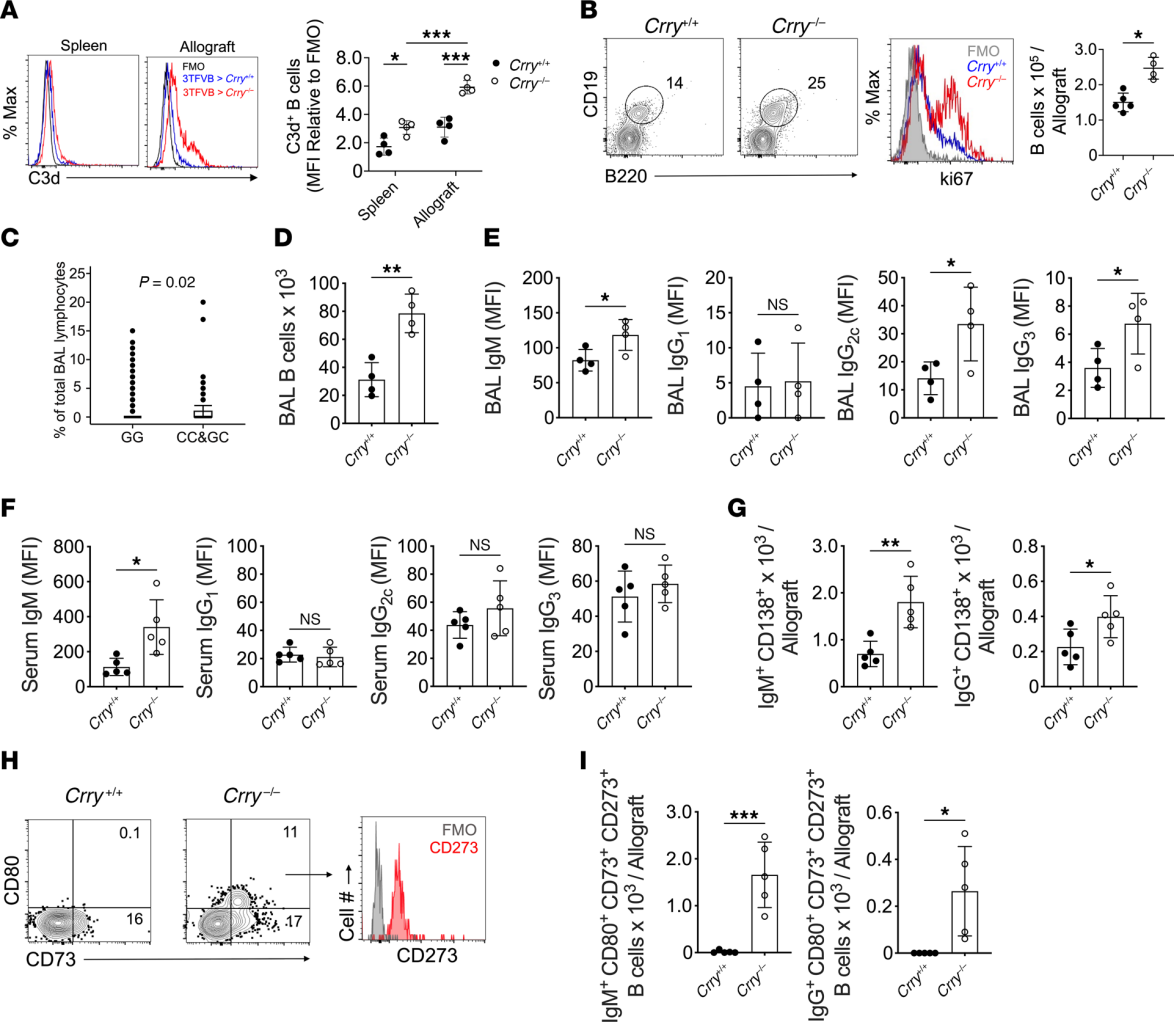
Enhanced intragraft B cell accumulation and activation in lung recipients with a defect in complement regulation. (**A**) Levels of C3d bound to B cells 2 days after the induction of DOX-induced allograft club cell injury. Representative FACS histogram of C3d B cell staining and (right panel) plots of C3d MFI normalized to a corresponding fluorescence minus one (FMO) control (*n* = 4/group). (**B**) POD 16 FACS contour plot of intragraft B cell abundance (left), ki67 histogram (proliferation) (middle), and a plot of total allograft B cell numbers on POD 16 (right). FACS contour plots and histograms are representative results from 4/group. (**C**) Percentages of BAL CD19^+^ lymphocytes in UCSF cohorts who carry the GG genotype and C3 R102G (CC&GC) polymorphisms. *P* = 0.02 by an unpaired *t* test. (**D**) Quantitation of BAL B cell numbers in POD 16 allografts (*n* = 4/group). Levels of POD 16 of (**E**) BAL (*n* = 4/group) and (**F**) serum (*n* = 5/group) donor reactive IgM, IgG_1_, IgG_2c_, and IgG_3_. (**G**) POD 16, intragraft CD138^+^ IgM^+^ and IgG^+^ Ab-secreting cell numbers (*n* = 5/group). (**H**) A representative FACS gating strategy used to identify intragraft CD80^+^CD73^+^CD273^+^ memory B cells, as shown in the bar graphs (**I**), which display the total intragraft numbers of these cells expressing either surface IgM (*n* = 5/group) or IgG (*n* = 4/group) on POD 16. Bar graphs and dot blots show mean ± SD values according to 2-way ANOVA with Šidák’s multiple comparisons test (**A**), and Welch’s *t* test (**B**, **D**–**G**, and **I**). *P < 0.05, **P < 0.01, ***P < 0.001.

Previous work has shown that activated C3 fragments bind to B cells to enhance their activation (48, 49). Given these observations, we asked whether Crry deficiency was associated with increased B cell activity in our CLAD model. C3d deposition on B cells was increased in the spleen and allografts of *Crry^–/–^* recipients (Figure 4A). There were also more total and proliferating CD19^+^B220^+^ B cells in the allografts of *Crry^–/–^* recipients (Figure 4B). We also quantified B cell levels in human LTx recipients carrying the C3 R102G variant (Figure 4C). A subcohort of 218 recipients with C3 R102G had increased CD19^+^ B cell frequencies in their BAL fluid after adjusting for age, sex, diagnostic group, and ethnicity. Likewise, we observed higher numbers of B cells in the BAL fluid of *Crry^–/–^* compared with wild-type recipients (Figure 4D and Supplemental Figure 2). De-novo DSAs are associated with an increased risk for CLAD development and progression (50). Therefore, we next measured DSA titers in *Crry^–/–^* recipients. Relative to wild-type recipients, *Crry^–/–^* recipients had higher IgM, IgG_2c_, and IgG_3_ DSAs in their BAL fluid (Figure 4E). By contrast, only IgM DSA numbers were elevated in the peripheral circulation of *Crry^–/–^* recipients (Figure 4F). Because DSA accumulation in BAL fluid indicated the local production of Abs, we assessed the abundance of Ab-secreting cells within allograft tissue. Analysis of allografts of *Crry^–/–^* recipients revealed more intragraft IgM^+^ and IgG^+^ Ab-secreting cells relative to wild-type recipients (Figure 4G and Supplemental Figure 3). CD73, CD86, and CD273 co-expression defines a memory B cell subset poised to become Ab-secreting cells (51). Strikingly, only *Crry^–/–^* recipient allografts had IgM^+^ and IgG^+^ CD73^+^CD80^+^CD273^+^ memory phenotype B cells (Figure 4, H and I).

**Figure 5:**
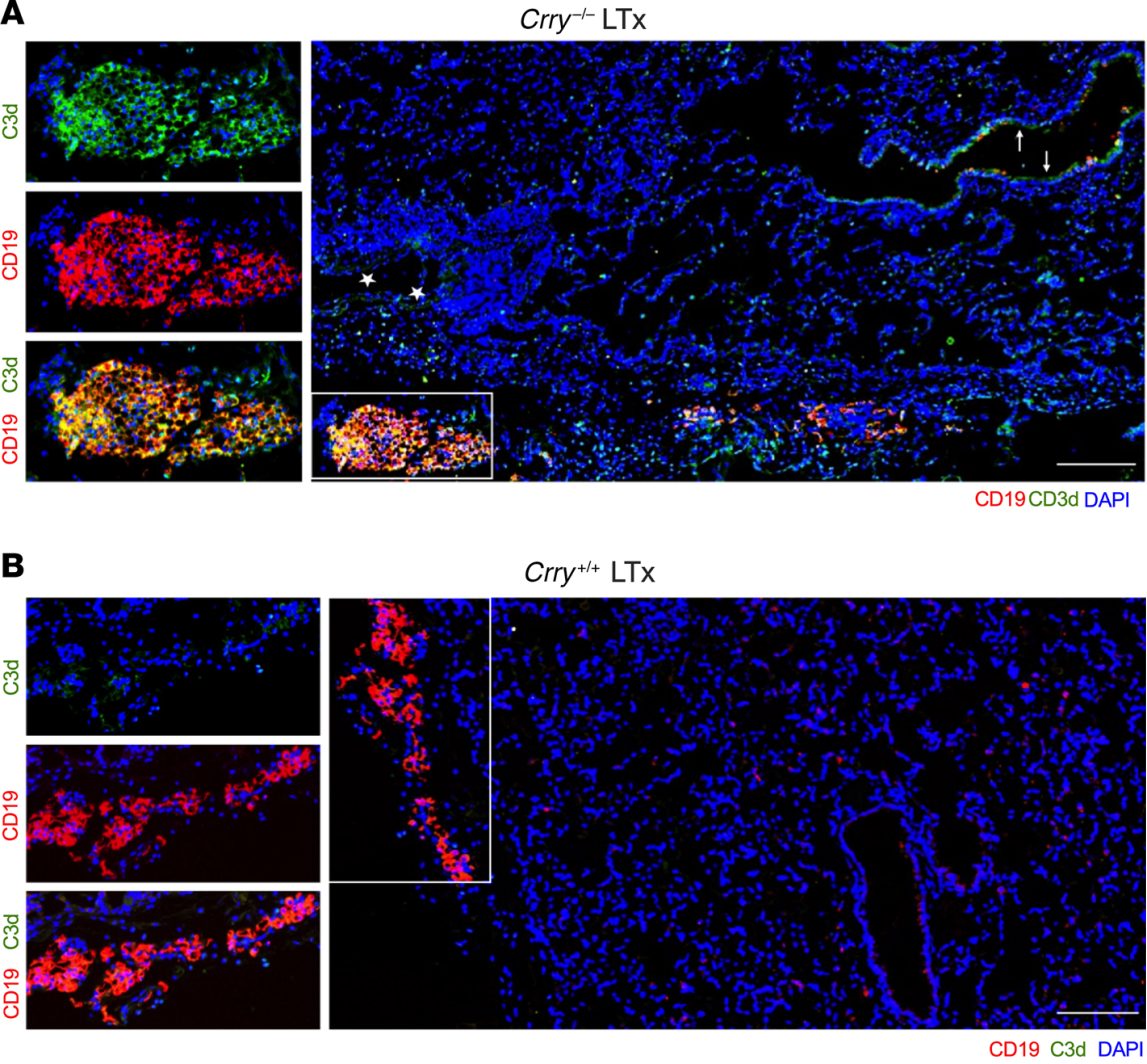
Lung allografts from Crry-deficient recipients show elevated C3d deposition. C3d and CD19 immunofluorescent staining of *Crry^–/–^* (**A**) and wild-type *Crry^+/+^* (**B**) recipient allograft tissue (LTx). Stars and arrows denote C3d staining on vascular endothelium and bronchial epithelium, respectively. Rectangular insets depict tertiary lymphoid aggregates enriched with B cells. Scale bars: 150 μm. The images shown are representative results of at least 3 transplants per group.

**Figure 6:**
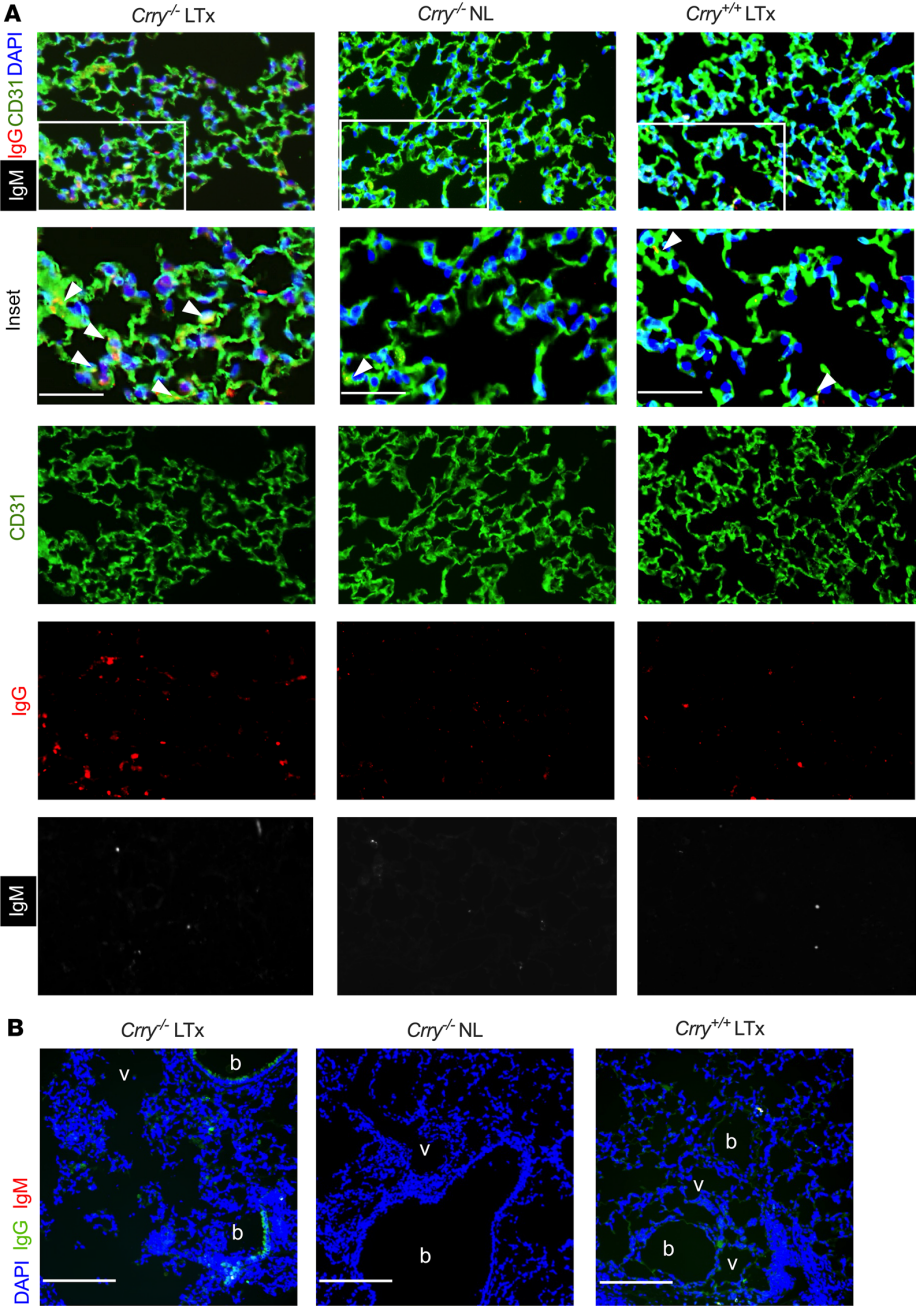
IgG deposition is more pronounced on lung allograft parenchyma from Crry-deficient recipients. (**A**) Immunofluorescence detection of IgG and IgM deposition on alveolar capillaries, as denoted by CD31 Ab staining. Inset scale bars: 25 μm. White arrowheads denote IgG deposition on alveolar capillaries. Images shown are representative of 4 transplants per group. (**B**) IgG and IgM deposition analysis on allograft bronchioles (b) and vessels (v). Inset scale bars: 150 μm. Images shown are representative of 4 transplants per group.

Given that our data indicated dysregulated complement regulation triggers the production of DSAs, we next sought evidence of intragraft classical pathway activation by assessing C3d deposition. Relative to wild-type lung recipients, C3d deposition was highly prevalent on B cells within tertiary lymphoid aggregates and, to a lesser degree, on the bronchial epithelium and vascular endothelium in allografts of *Crry^–/–^* recipients (Figure 5, A and B). We also analyzed Ab deposition in the lung parenchyma. IgG deposition was more apparent on the allograft alveolar capillaries and bronchial epithelium of *Crry^–/–^* recipients relative to the native lungs of *Crry^–/–^* recipients or wild-type recipient allografts (Figure 6, A and B). Although IgM expression was present on cells within the pleural cavity, IgM deposition was almost undetectable on lung parenchyma (Supplemental Figure 4).

**Figure 7:**
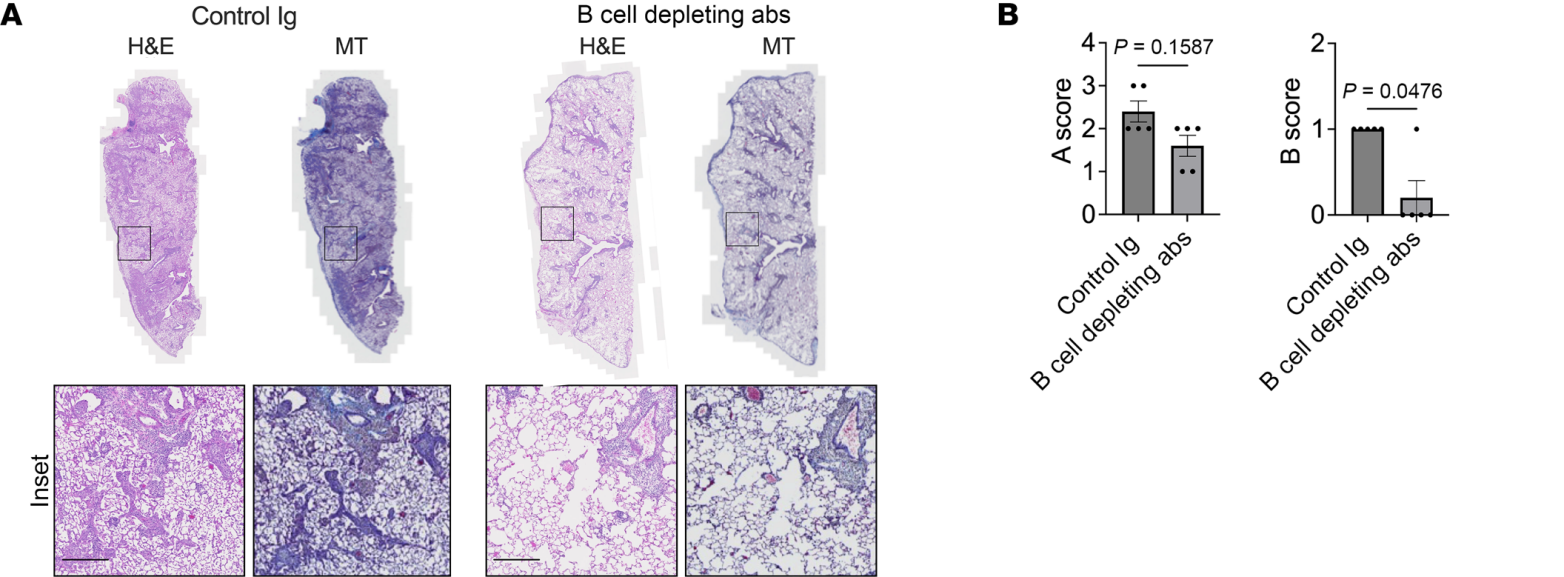
CLAD development in Crry-deficient allograft recipients is driven by B cells. (**A**) Crry-deficient lung recipients received B cell–depleting Abs or control Ig on PODs 6 and 12 and were evaluated for allograft injury on POD 16. Inset scale bars: 200 μm. The histology shown is a representative result of H&E and Masson’s trichrome (MT) allograft staining of 5 transplants per group. (**B**) POD 16 blinded A (vascular) and B (airway) ISHLT consensus pathological scoring (*n* = 5/group). The bar graph shows mean ± SD; the Mann-Whitney *U* test was used to generate *P* values.

Finally, to examine if B cells are required for CLAD development in our model, *Crry^–/–^* recipients were treated with B cell–depleting Abs and evaluated on POD 16. Compared with recipients that received control Abs, B cell depletion significantly inhibited pathological signs of CLAD development (Figure 7, A and B, and Supplemental Figure 5).

## Discussion

CLAD is characterized by a persistent and irreversible decline in lung function and is the major cause of long-term morbidity and mortality after LTx (2, 52). Both innate and adaptive immune responses contribute to the progression of CLAD (53). The complement system is an early component of the innate immune response and has been shown to influence outcomes in primary graft dysfunction, a form of acute lung injury occurring early after LTx (54–56). However, complement activation also influences long-term adaptive immune responses in multiple organ systems and has been implicated in CLAD pathogenesis (9). Complement activation has been shown to augment alloimmune CD4^+^ and CD8^+^ T cell responses through C3a-C3aR interactions (21, 57, 58). More specifically, IL-17 activation in lung allografts suppresses membrane regulatory protein expression in airway epithelial cells and results in increased local complement activation (9). Increased C3a levels enhances IL-17 production, setting up a feed-forward loop that worsens obliterative bronchiolitis. Increased C3a levels also upregulate TGF-β, a key mediator of obliterative bronchiolitis, which downregulates membrane regulatory proteins such as CD46/Crry and CD55 in models of lung fibrosis (23, 59). This TGF-β–mediated loss of regulatory proteins is abrogated by both C3aR and C5aR1 inhibition, demonstrating how complement activation is a key component of amplification loops propagating irreversible airway and parenchymal fibrosis (23, 59).

There exists considerable variability in the onset of CLAD. Risk factors such as infection, air pollution, aspiration, and a prior history of primary graft dysfunction or acute cellular rejection (ACR) predispose to an earlier onset of CLAD (1, 6, 60). Given the heterogeneity of immune responses, it has been increasingly recognized that particular recipients may also be genetically predisposed to worse CLAD-free survival (61, 62). The C3 R102G functional polymorphism has also been associated with worse outcomes in kidney and liver transplantation (63, 64). LTx recipients who had a donor with C3 R102G had worse bronchiolitis obliterans syndrome–free survival (65). However, at least in renal transplantation, there has been no benefit to matching donors with recipients, based on this polymorphism (66), and long-term LTx outcomes in recipients with advanced lung disease and C3 R102G have not been investigated to date. Surprisingly, we observed that nearly one-third of LTx recipients harbored C3 R102G polymorphism, which exceeds the minor allele frequency of 10% to 15% in the general population, suggesting it may also play a role in the pathophysiology of the underlying lung disease. However, having such a common polymorphism among recipients also necessitates a better understanding of how it affects CLAD-free survival in the setting of impaired complement regulation. Moreover, the prevalence of this functional polymorphism affords avenues to test whether modulating complement activation in LTx recipients with this polymorphism may facilitate personalized immunomodulation to improve CLAD-free survival.

To further probe the effects of dysregulated complement activation on CLAD development, we analyzed graft injury and inflammation in an orthotopic mouse LTx model using *Crry^–/–^* mice as recipients. Crry is a membrane complement regulator expressed on a wide variety of murine cells that prevents C3 fragment deposition in response to classical or alternative complement pathway activation (67). Like CD46, Crry regulates complement activation through Factor I–mediated cofactor activity (38, 39). In contrast to wild-type recipients, *Crry^–/–^* recipients had considerably more evidence of graft airway injury and higher levels of DSAs, suggesting that CLAD results from Ab-mediated complement activation. However, whether DSA-mediated complement activation is required for graft epithelial injury remains controversial. Introducing either complement-activating or non–complement-activating IgG against donor antigens into a heterotopic mouse tracheal transplant model has been shown to generate obliterative airway disease (68). In the orthotopic mouse LTx model, DSA generation occurred de novo approximately 8 days after club cell injury.

Given that our data indicated dysfunctional complement regulation triggers the production of DSAs, we next sought evidence of intragraft classical pathway activation by assessing C3d deposition. We observed more C3d deposition in *Crry^–/–^* compared with wild-type recipient allografts, with most activity concentrated on B cells within tertiary lymphoid aggregates and, to a lesser extent, on the vascular endothelium and bronchial epithelium. In contrast, C3d deposition was nearly absent from allografts of wild-type recipients. Additionally, IgG deposition was more apparent on the allograft alveolar capillaries and bronchial epithelium in the setting of impaired complement regulation in recipients. Although IgM expression was present on cells within the pleural cavity, IgM deposition was almost undetectable on lung parenchyma. Finally, we showed that B cell depletion significantly inhibited pathological signs of CLAD development in our animal model. Hence, despite the intrinsic differences in the animal model of Crry deficiency compared with the C3 polymorphism in humans, the work aids us in deciphering how impaired complement regulation drives chronic rejection after LTx. Specifically, the C3 polymorphism in humans results in impaired complement regulation as the charge of the C3 protein synthesized in the setting of the R102G polymorphism impairs the ability of the regulatory protein Factor H to inactivate it (36). Recipients harboring this polymorphism had worse CLAD-free survival in the presence of DSAs and had increased numbers of CD19^+^ B cells in their BAL. In the mouse model, Crry deficiency also resulted in impaired regulation and increased C3 activation (67), thereby leading to accelerated CLAD via a B cell–dependent process. Taken together, these data point to potential pathways by which impaired complement regulation enhances humoral immunity in the setting of LTx.

Increased complement activation has been reported to enhance B cell responses via multiple pathways (69). For example, resting B cells express high amounts of the complement receptor type 2 (CR2/CD21), which binds antigen-bound C3 fragments (48, 70). Co-clustering CR2 with the B cell receptor lowers the threshold of B cell activation, leading to enhanced Ab production (71). CR2 is also expressed on stromal cells and follicular dendritic cells, where it plays a vital role in antigen retention to promote the generation of B cell memory and Ab-secreting cells (72). In this regard, we noted C3d deposition on B cells within tertiary lymphoid aggregates within *Crry^–/–^* allografts. Our previous work has demonstrated that germinal-center B cells primarily housed within intragraft tertiary lymphoid tissues play a crucial role in the local production of IgG, but not IgM DSAs, while also promoting the generation of memory B cells and Ab-secreting cells within allograft tissue (73). This raises the possibility that our observation of BAL DSA accumulation in *Crry^–/–^* recipients is due to complement-mediated activation of germinal-center B cells (74). To this end, previous work has shown that complement fragment deposition on B cells promotes their activation (75). The B cell receptor C1q binding domain drives the fixation of C3 fragments to the B cell membrane and generates optimal antigen-specific responses (49). Relative to these previous reports, we observed increased B cell accumulation, enhanced proliferative responses, and elevated levels of memory B cells and Ab-secreting cells in allografts after transplantation into *Crry^–/–^* recipients. Interestingly, a recent single-cell RNA-sequencing study revealed an association between intragraft accumulation of plasma cells and CLAD in human and mouse allografts (15). Collectively, data from the orthotopic mouse LTx model implicate dysregulated complement activation in stimulating local B cell activation responses that promote CLAD development.

Observations from the animal model prompt questions as to whether the increased susceptibility to CLAD in the setting of the C3 polymorphism is due to B cell–dependent pathways. We observed an increased proportion of BAL CD19^+^ B cells in recipients with the polymorphism. Moreover, CLAD-free survival in the setting of C3 R102G polymorphism occurred primarily in patients who developed anti-HLA donor-specific Abs, which are B cell dependent. Hence, although DSA prevalence did not differ among those with and without the polymorphism, we attribute these observations to inherent differences in investigating Ab-mediated alloimmune responses between mice and humans. Our categorization of DSAs in patients is based on MFI from a bead-based platform, whereas we measure titers in the mouse model. Other possibilities include contributions from non-HLA Abs (12) and DSAs deposited in the allograft (76, 77). Knowing that RAS is a form of CLAD associated with humoral immune responses and worse outcomes, we also interrogated if patients with the C3 polymorphism had an increased odds of RAS using forced vital capacity as a prognostic adjunct (78), but we did not observe a difference. Moreover, because the B cell enumeration in our patient cohort was done using a clinical flow cytometry panel (79) and we do not have banked BAL samples for this cohort, we were unable to conclusively link the increased BAL CD19^+^ B cells in our recipients with the polymorphism to B cell activation. Yet, plasma from patients harboring this polymorphism has been shown to activate the alternative pathway (AP) more efficiently, and these patients demonstrate increased AP amplification (36). This increased activation and amplification would generate more complement activation fragments (e.g., C3d), which would bind to receptors such as CR2, thus enhancing B cell responses, including DSA production. Anti-HLA Abs have also been shown to induce epithelial cell proliferation as well as induce the production of soluble growth factors, thus perpetuating injury that leads to CLAD (80, 81). Thus, the worse CLAD-free survival in patients with post-transplant DSAs harboring the C3 R102G polymorphism, and the enrichment of BAL CD19^+^ B cells, encourage us to build on our observations from the animal model of Crry deficiency in recipients that demonstrated high BAL C3d^+^ B cells, elevated levels of intragraft DSAs, and B cell–dependent CLAD development. Our data collectively suggest that CLAD development in the setting of dysregulated complement activation is mediated by increased humoral responses. Future work would involve measuring markers of complement activation in the BAL of recipients harboring this polymorphism, improved subphenotyping of B cells and their subsets to assess for complement-mediated activation in longitudinal samples from LTx recipients, as well as staining lung tissues with C3d and IgG, as we have demonstrated in our animal model, to conclusively prove that the C3 R102G polymorphism increases the risk of CLAD via B cell activation.

There are also other potential mechanisms by which complement activation may be increasing CLAD risk, including complement activation–driven T cell effector responses that could indirectly influence B cell responses, increased Ab-dependent cellular cytotoxicity, and direct toxicity through HLA ligation that drives fibrosis (82, 83). From the standpoint of leveraging this polymorphism for patient selection in future clinical trials, mechanistic studies could involve determining whether the C3 R102G mutation worsens allograft injury via a C3d-CR2–mediated axis, perhaps by promoting memory B cell responses. Further investigation into probing impaired classical pathway activation on allograft endothelial surfaces could be addressed with inhibitors of C1-esterase, C3, or C5. Given the availability of FDA-approved anticomplement agents that target these molecules, such therapies could be considered for complement-mediated injury in LTx recipients (16).

Our study has limitations, including insufficient sample sizes at both sites to test whether this polymorphism predisposes to AMR as a mediator of CLAD. Additionally, we did not observe significant differences in the prevalence of ACR or lymphocytic bronchiolitis in either the BJH or the UCSF cohort, regardless of the polymorphism. However, we are cautious about overinterpreting ACR data in human recipients, because pathologist agreement is low, protocols differ between centers, and ACR is a heterogeneous process prone to sampling error (84, 85). Moreover, many patients with ACR do not develop CLAD, and many recipients with CLAD have never experienced prior ACR (11, 86, 87). Emerging data suggest ACR and AMR may coexist within the same allograft and be mechanistically linked (88, 89). Because we cannot exclude the possibility that the C3 polymorphism drives ACR and lymphocytic bronchiolitis, we elected not to include them in the final multivariable models, to prevent collider bias. We also do not routinely measure other non-HLA anti-allograft Abs in the allograft or circulation. However, we observed that IgG2c and IgG3 (but not IgM or IgG1) levels were elevated in the BAL of *Crry^–/–^* recipients as compared with in their serum, whereas only IgM was elevated in the serum of *Crry^–/–^* recipients compared with wild-type allograft recipients. Along these lines, although IgG1 strongly activates complement in humans, IgG2 subclasses and IgG3 strongly activate complement in mice (90–92). These observations support the role of local Ab production and intragraft complement-mediated responses in LTx recipients as downstream responses to impaired complement regulation. Nevertheless, despite observing the propensity toward CLAD in both humans and mice, we acknowledge our modeling of impaired complement regulation in mice is not entirely analogous to mechanisms that operate in humans (93). Another limitation is that collagenase digestion was used to generate allograft single-cell suspensions, which could cause CD138 shedding, leading to an underestimate of Ab-secreting cell numbers (94). Although we did not include isograft data in our experiments, we have previously reported that syngeneic transplants, in which 3T-B6 lungs were engrafted into B6 recipients, did not develop chronic rejection despite extensive club cell injury (44). Additionally, because there is no validated scoring system for the pathology of CLAD in mouse models, we used the B score for histopathological scoring of airway involvement (95). Moreover, we used Masson’s trichrome staining and tissue hydroxyproline assays as a surrogate for the fibrosis that occurs in chronic rejection. The proportion of recipients with RAS was low to draw any meaningful conclusions, and we did not have total lung capacity data on our recipients in these cohorts. However, whereas increased humoral responses are associated with RAS, DSAs remain a risk for obstructive CLAD (96–98) and can affect lung function even in the absence of clinical AMR (99). Future clinical studies should focus on determining if complement-mediated humoral immune responses contribute to RAS (14).

Using an orthotopic mouse model of LTx, we show that dysregulated complement regulation promotes CLAD through enhancing B cell activation. In a multicenter study, we demonstrate worse CLAD-free survival in recipients who carry the C3 R102G polymorphism and have DSA. The mechanistic basis for this finding may primarily be B cell dependent, suggesting that conventional T cell immunosuppression regimens may be of limited efficacy at preventing CLAD in patients with the C3 R102G polymorphism. By describing a sizeable subgroup of LTx recipients with a polymorphism that predisposes them to impaired complement regulation, we identify a cohort that can form the basis of clinical trials in personalized immunomodulation to improve long-term outcomes after LTx.

## Methods

### Sex as a biological variable.

Our study investigated data from both male and female human participants, and similar findings are reported for both sexes. Our orthotopic LTx model used male donor lungs transplanted into male recipients or female donor lungs transplanted into female recipients.

### Study design, settings, and participants.

We performed a retrospective cohort study and screened 1,218 primary LTx recipients at Washington University/BJH between January 1, 2005, and December 31, 2022, with follow-up through December 31, 2023. We excluded those who did not consent (*n* = 250) and those for whom genotyping had not been performed (*n* = 576) (Figure 1A). We also included 864 primary LTx recipients at UCSF through September 30, 2021. We excluded those who did not consent (*n* = 153) and those for whom genotyping had not been performed (*n* = 293) (Figure 1B).

### Clinical variables.

Clinical management at these 2 centers has been described in prior studies (11, 61). HLA Abs were detected using the LABScreen Single Antigen assay, and DSA testing was considered positive if the reported MFI was ≥2,000. The time from transplantation to the first detection of DSA after transplantation was defined as time to DSA positivity. A positive bacterial culture from a bronchial wash or BAL was defined as bacterial isolation. Primary graft dysfunction and ACR were defined based on ISHLT criteria. CLAD was defined as a persistent decline in forced expiratory volume in 1 second ≤80% for at least 3 weeks without a specific cause. CLAD-free survival was defined as the earliest occurrence of either CLAD or death, whichever came first.

### Genotyping.

At BJH, salivary specimens from consented patients were genotyped for the C3 R102G (*rs2230199*) polymorphism, based on the TaqMan assay, as previously described (100). At UCSF, blood specimens were genotyped using a transplant-targeted Affymetrix gene array designed by the iGeneTRAiN consortium that directly typed the C3 R102G (*rs2230199*) polymorphism (101). At both centers, recipients with CC/CG (minor allele) were compared with those with GG.

### BAL analysis.

Specimens included in this study were from bronchoscopies conducted as part of routine clinical management, when patients had an unexplained decline in their lung function and were being evaluated for CLAD. In the UCSF cohort, bronchoscopies were performed for clinical indication or for allograft surveillance at 0.5, 1, 2, 3, 6, 12, 18, and 24 months after transplantation. BAL cell subsets, including B cells, are routinely quantified by clinical cytometry at UCSF.

### Crry^–/–^ mice.

Crry deficiency has previously been reported as being embryonic lethal because breeding *Crry^+/–^* mice together failed to achieve any *Crry^–/–^* mice (102–104). However, strategic breeding procedures led to the generation of *Crry^–/–^* mice. In detail, breeding *Crry^+/–^C3^–/–^* mice resulted in *Crry^–/–^C3^–/–^* offspring, indicating that *Crry^–/–^* mice can survive in the condition of C3 deficiency. We then bred *Crry^+/–^* males with *Crry^–/–^C3^–/–^* females to generate *Crry^–/–^C3^+/–^* mice. These mice only have half capacity of the AP activity (39), so we predicted that mothers with the genotype of *Crry^–/–^C3^+/–^* would have insufficient AP activity to attack embryos that had a *Crry^–/–^* genotype. Considering this, we bred *Crry^+/–^* males with *Crry^–/–^C3^+/–^* females, leading to the generation of *Crry^–/–^C3^+/+^* pups, which are *Crry^–/–^* but C3 sufficient. BAL was performed on these mice by instilling 1 mL of PBS plus enzyme inhibitor (Halt Protease and Phosphatase Inhibitor Single-Use Cocktail; Thermo Fisher, catalog 78442) into both lungs, performing centrifugation at 500*g* for 5 minutes, followed by aliquoting the supernatant.

### Immunoblot analysis.

C3 was detected using immunoblotting of BAL and serum (1:3,000; MP Biomedicals, catalog 55444). BAL supernatant (3 μL) was mixed with 7 μL of PBS, resulting in a total volume of 10 μL. Subsequently, 3.5 μL of 4× reducing buffer (1:10 ratio of β-mercaptoethanol added to Laemmli buffer; Bio-Rad, catalog 1610747) was added to the diluted samples, bringing the final volume to 13.5 μL. The samples were vortexed for 5 seconds and then centrifuged. After centrifugation, the samples were incubated in a heating block at 98°C for 6 minutes. After this heating step, the samples were centrifuged again before being loaded onto the gel.

For serum samples, 1 μL of each sample was combined with 9 μL of PBS, also resulting in a total volume of 10 μL. Like the BAL samples, 3.5 μL of 4× reducing buffer was added, and the samples were vortexed, centrifuged, heated, and centrifuged again before gel loading. Purified C3 protein was prepared at a final concentration of 7.5 ng/μL by diluting 1 μL of the purified protein in 99 μL of PBS, followed by the addition of 33.5 μL of 4× reducing buffer.

### Orthotopic left LTx.

C57BL/6 (B6) mice were purchased from The Jackson Laboratories. Club cell secretory protein (CCSP) rtTA/TetOCre/DT-A mice on a mixed background were initially generated by Jeffrey Whitsett of The Children’s Hospital of Cincinnati. These mice were then backcrossed by our group to greater than 99% onto a FVB/J background using microsatellite-assisted accelerated backcrossing (MAX-BAX; Charles River) to generate 3T-FVB left lung donors (44). Left lung orthotopic LTx has been described (105).

To induce allograft acceptance, recipients received 250 μg of CD40L Abs (clone MR1) i.p. on POD 0 and 200 μg of human recombinant CTLA4 Ig on POD2 (106). Club cell injury was triggered by DOX ingestion via food (625 mg/kg chow; ENVIGO) and water (2 mg/mL; Doxycycline hyclate, MilliporeSigma) for 30 hours to induce club cell injury. To deplete B cells, rat anti–mouse CD19 (clone 1D3; Bio-X-Cell), rat anti–mouse B220 (clone RA3.3A1/6.1; Bio-X-Cell), and mouse anti–mouse CD22 (clone CY34.1; Bio-X-Cell) (150 μg/mouse for each) were administered i.p. on PODs 6 and 12. Control recipients received 450 μg of rat IgG1 isotype control (clone TNP6A7; Bio-X-Cell). After 1 day, recipients were injected with mouse anti–rat IgG (clone Mar-18.5; Bio-X-Cell) at 150 μg/mouse.

### Immunohistological staining.

Harvested grafts were formaldehyde fixed, paraffin embedded, and stained with H&E or Masson’s trichrome stain. LTx histology was graded by a blinded pathologist using the 2007 revision of the 1996 ISHLT working formulation for the standardization of nomenclature in the diagnosis of lung rejection (45). For immunohistochemical analysis, paraffin sections were first blocked with 5% goat serum and 2% fish gelatin (both from Sigma-Aldrich) at 25°C for 45 minutes. Sections were then stained with 1:500 polyclonal rabbit anti–mouse/rat CCSP (catalog WRAB-3950, Seven Hills Bioreagents) and mouse anti-acetylated tubulin (1:5,000; clone 6-11 B-1; Sigma-Aldrich) overnight at 4°C. For secondary Ab-mediated immunofluorescent visualization, we used 1:1,000 goat anti–mouse Alexa Fluor 488–labeled secondary Abs (catalog A-11-001, Thermo Fisher), 1:1,000 donkey anti–goat Alexa Fluor 488 (catalog A-11055, Thermo Fisher), 1:1,000 donkey anti–rabbit Alexa Fluor 555 (catalog A-31572, Thermo Fisher), and 1:1,000 goat anti–rabbit Alexa Fluor 555 (catalog 4413S, Cell Signaling Technology). To assess C3d tissue deposition, 1:100 goat-anti–mouse/rat C3d (Bio-Techne), 1:100 rabbit anti–goat IgG-biotin (catalog 31172, Thermo Fisher), dilutions were used and stained with VectorStain ABC-HRP kit (catalog PK-4000, Vector) according to the manufacturer’s recommendations. To detect IgM and IgG adsorption to lung parenchyma, allografts were first subjected to three 1.0 mL PBS BAL extractions, flushed vascularly to remove leukocytes and nonspecifically bound immunoglobulin, paraffin embedded, and then stained with 1:250 anti-mouse CD31-FITC (clone 330, Thermo Fisher), 1:100 goat anti–mouse IgG-PE (catalog 31861, Thermo Fisher) or 1:100 goat anti–mouse IgG-FITC (catalog 31457, Thermo Fisher), and 1:100 goat anti–mouse IgM-DyeLight 650 (catalog SA5-10153, Thermo Fisher) or 1:100 goat anti–mouse IgM-Alexa Flour 555 (catalog A-21426, Thermo Fisher).

### Hydroxyproline analysis.

To determine intragraft collagen concentrations, a Hydroxyproline Assay Kit (catalog MAK569, Sigma-Aldrich) was used according to the manufacturer’s recommendations. Allograft tissue was extracted for protein with 6.0 N HCl for 3 hours at 120°C and dried at 60°C overnight. Then, 10 μg of protein was assayed for reactivity for oxidized hydroxyproline with 4-(dimethylamino) benzaldehyde, which yields a colorimetric product that was quantified by absorbance at 560 nm on a Biotek Synergy HTX Microplate Analyzer.

### Flow cytometric analysis.

Lung tissue was minced and digested in an RPMI 1640 solution with type 2 collagenase (50 μg/mL; Worthington Biochemical Corp.) and 5 units/mL DNAse (MilliporeSigma) for 30 minutes at 37°C and then filtered through a 70 μm cell strainer (Thermo Fisher) and treated with ammonium-chloride-potassium lysing buffer (Worthington Biochemical Corp.). Live cell discrimination was conducted with Zombie Fixable Dye (BioLegend). Cell surface staining was performed with the following Abs: CD45 (clone 30-F11; eBioscience), CD45.2 (clone 104; BioLegend), CD90.2 (clone 53-2.1; eBioscience), CD4 (clone RM4-5; eBioscience), CD8α (clone 53-6.7; eBioscience), B220 (RA3-6B2; Biolegend), CD19 (1D3; BD Biosciences), rabbit anti–human/mouse/rat C3d (catalog bs-4877R; Bioss), CD138 (281-2; Biolegend) CD38 (S21016F; Biolegend), IgD (11-26c.2a; Biolegend), Foxp3 (FJK-16s; Thermo Fisher), CD73 (TY/11.8; Biolegend), CD80 (16-10A1; Biolegend), CD273 (TY25; Thermo Fisher), CD31 (clone 390; BioLegend), CD34 (clone HM34; BioLegend), and CD326 (clone G8.8; BioLegend). Intracellular CCSP (Seven Hills Bioreagents) and polyclonal goat anti–mouse IgM heavy chain (SouthernBiotech) staining was conducted with the Cytofix/Cytoperm kit (BD Biosciences) following the manufacturer’s recommendations. Staining for Foxp3 (FJK-16s; eBioscience) and Ki-67 (16A8; BioLegend) was conducted with the Intranuclear Transcription Factor Staining Buffer Kit (Invitrogen) per the manufacturer’s recommendations. For IFN-γ and IL-17A expression, cells were first stimulated with 1 μM ionomycin (MilliporeSigma) and 20 ng/mL PMA (MilliporeSigma) for 3.5 hours, with 2 μM Golgi Plug (BD Biosciences) added for the last 3 hours of stimulation. Cells were then intracellularly stained with IFN-γ (clone XMG1.2; eBioscience) and IL-17A (clone TC11-18H10.1; BioLegend) using a Cytofix/Cytoperm kit. For DSA analysis, FVB splenocytes were resuspended at 4 × 10^6^ cells/mL in FACS buffer (PBS, 0.1% BSA, 0.02% NaN_3_) and treated with FcγR block on ice for 10 minutes (clone 93; Biolegend). BAL fluid was diluted to 1:15 in PBS, and serum was diluted to 1:25 in PBS, and 50 μL of these diluted specimens was mixed with 50 μL of FVB splenocytes, which were then incubated for 1 hour at 4°C. Cells were washed twice with FACS buffer and stained with goat anti–mouse IgM heavy chain-APC (catalog 1020-11S, SouthernBiotech), goat-anti–mouse IgG_2c_-AlexaFluor 647 (catalog 1079-3, Southern Biotech), goat-anti–mouse IgG_1_–Alexa Fluor 488 (catalog 115-545-071, Jackson ImmunoResearch), and goat anti–mouse IgG or goat anti–mouse IgG_3_ Alexa Fluor 488 (catalog A-21151, Thermo Fisher), and then washed twice more before FACS-based MFI analysis.

### Statistics.

Data were analyzed by the Mann-Whitney *U* test or 1-way ANOVA and are represented as mean ± SD. Statistical analysis was conducted in R (version 4.3.2) and GraphPad Prism software, version 9.0. *P* < 0.05 was considered significant. Time-to-event analyses were performed using Kaplan-Meier curves with log-rank test for equality. Multivariable analyses were conducted via Cox proportional hazards regression.

### Study approval.

Animal experiments were conducted in accordance with an approved IACUC protocol (Washington University, 19-0827). The BJH participants provided written informed consent in accordance with the Washington University School of Medicine Institutional Review Board for Human Studies protocol (no. 201811073 and 201105421). The UCSF participants provided written informed consent in accordance with the Institutional Review Board for Human Studies protocol (no. 13-10738).

### Data availability.

Values for all data points in graphs are reported in the Supporting Data Values file. The study participants have not consented to sharing genetic data publicly; therefore, the data cannot be uploaded to a publicly accessible server. Deidentified data are provided using a material transfer agreement/data use agreement with the institutions.

## Author contributions

HSK, DRC, JRG, and AEG designed the studies. HSK, FL, ZL, AT, LG, DHK, XW, and AEG conducted the experiments. HSK, LKT, DRC, FL, ZL, LG, NRS, CC, HJH, CAW, RRH, DZ, YT, and VED acquired the data. HSK, LKT, DRC, CTB, and AEG analyzed the data. XW, DHK, DEB, DK, and JPA provided reagents. HSK, LKT, DRC, LG, XW, JRG, and AEG wrote the manuscript. HSK, LKT, DRC, DK, JPA, JRG, and AEG acquired funding for this work.

## Funding support

This work is the result of NIH funding, in whole or in part, and is subject to the NIH Public Access Policy. Through acceptance of this federal funding, the NIH has been given a right to make the work publicly available in PubMed Central.

- NIH grants R01-HL166449, R01-HL169860, and R35-GM136352 to HSK.
- Children’s Discovery Institute to HSK.
- Longer Life Foundation to HSK.
- NIH grant K01-HL155231 to LKT.
- Robert Wood Johnson Foundation to LKT.
- Doris Duke Charitable Foundation to LKT.
- VA Office of Research and Development grant BX005301 to DRC.
- Cystic Fibrosis Foundation grant to CALABR22G0 DRC.
- NIH grants P01-AI116501 and R01HL094601 to DK.
- US Department of Veteran’s Affairs grant 1I01BX002730) to DK.
- Cystic Fibrosis Foundation grant KREISE24AB0-CLAD to DK.
- Foundation for Barnes-Jewish Hospital to DK.
- NIH grant R35-GM136352 to JPA and XW.
- NIH grants R01-HL161048, R01-HL151552 to JRG.
- Cystic Fibrosis Foundation grant GREENL21AB0 to JRG.
- VA Office of Research and Development grant CX002011 to JRG.
- NIH grants P01-AI116501, R01HL167277, R01HL094601, R01HL157407, and P41EB025815 to AEG.
- Cystic Fibrosis Foundation grant GELMAN25XX0 to AEG.
- The Barnes-Jewish Foundation to AEG.

## Supplementary Material

Supplemental data

Unedited blot and gel images

Supporting data values

## Figures and Tables

**Table 4:**
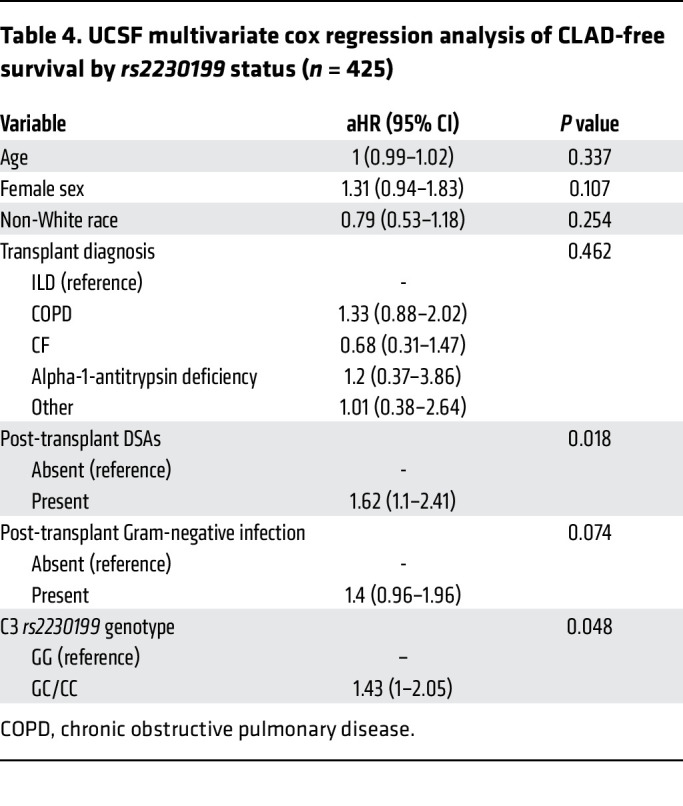
UCSF multivariate cox regression analysis of CLAD-free survival by *rs2230199* status (*n* = 425)

**Table 3:**
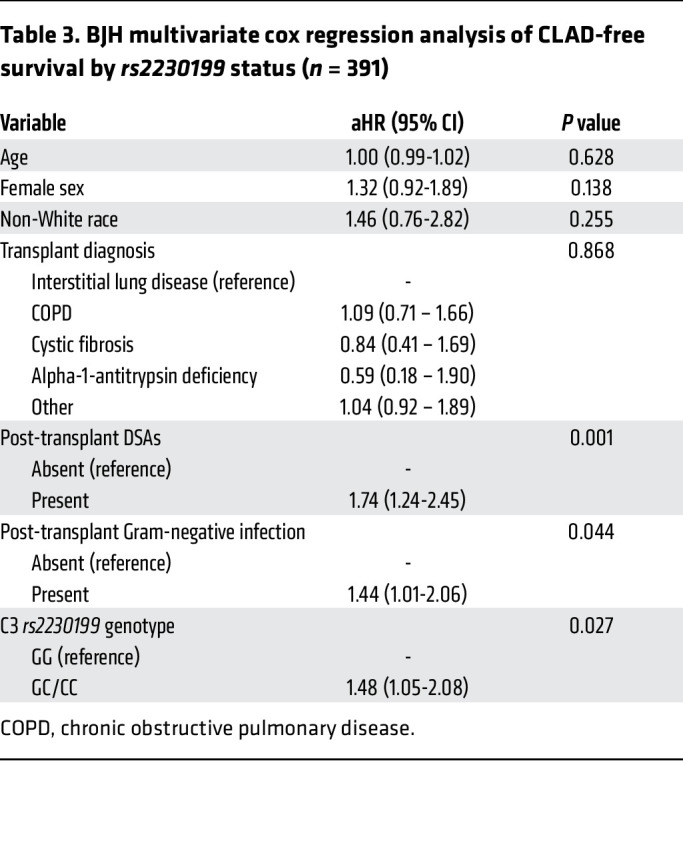
BJH multivariate cox regression analysis of CLAD-free survival by *rs2230199* status (*n* = 391)

**Table 2:**
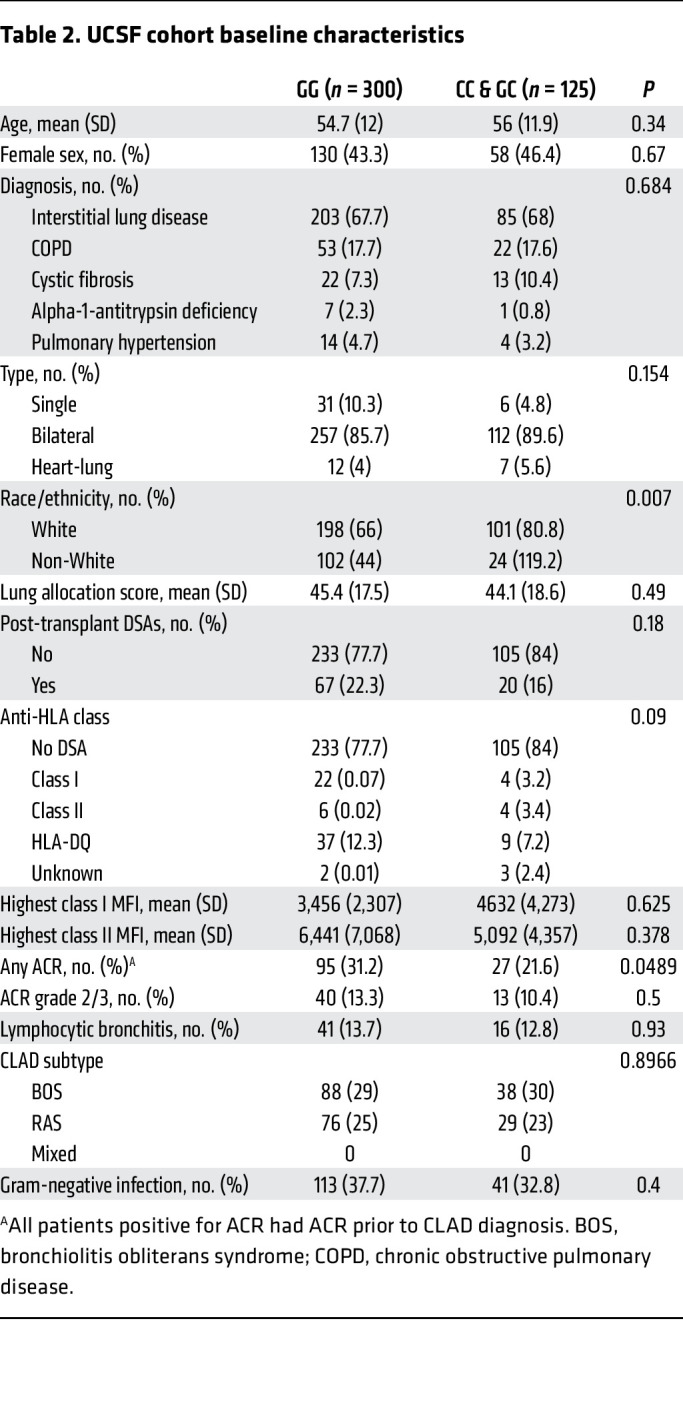
UCSF cohort baseline characteristics

**Table 1:**
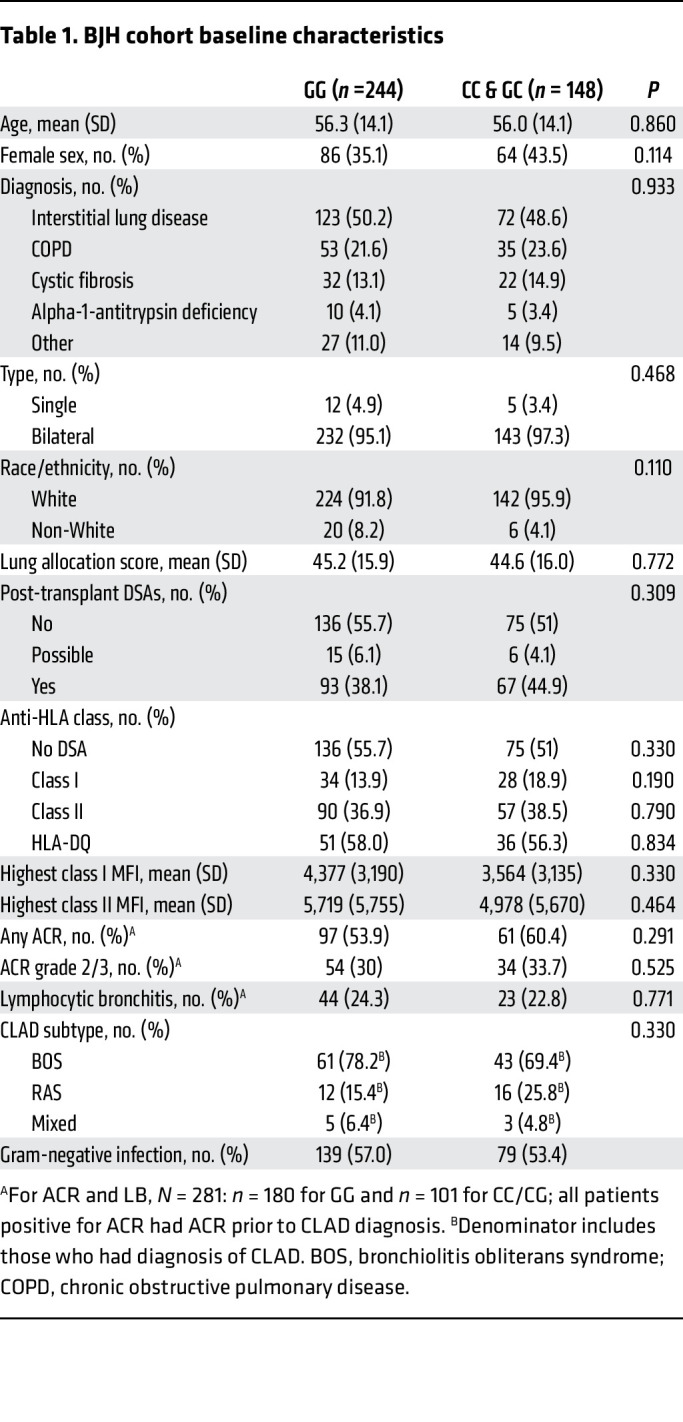
BJH cohort baseline characteristics

## Version 1 11/11/2025

In-Press Preview

## Version 2 01/02/2026

Electronic publication
