# Supplementary material for: Impaired complement regulation drives chronic lung allograft dysfunction after lung transplantation
Source: J Clin Invest. 2025 Nov 11;136(1):e188891. doi: 10.1172/JCI188891 (PMC12721912; doi:10.1172/JCI188891)
Supplement: Supplemental data [file jci-136-188891-s330.pdf]

Supplemental Figure 1

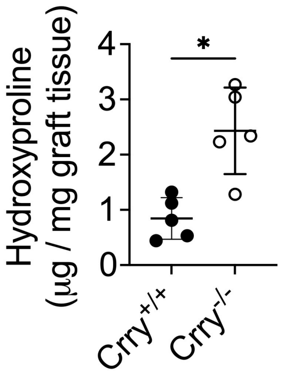

Supplemental Figure 1: Allografts of Crry-deficient recipients accumulate collagen. Wildtype and Crry-deficient recipients of lung allografts were evaluated for hydroxproline content on POD 16 (N=5/group). The dot blot shows mean levels ± standard deviation for a Welch's t-test, where *p <0.05.

Supplemental Figure 2

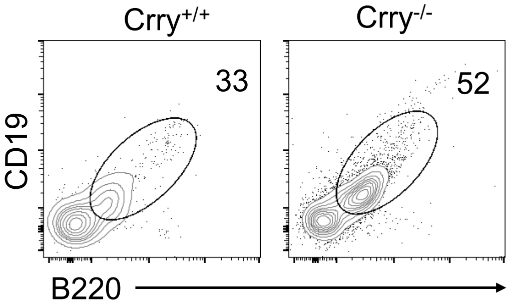

Supplemental Figure 2: Crry-deficient lung allograft recipients have high levels of airspace B cells. POD 16 BAL of wildtype and Crry-deficient recipients were analyzed for B cell percent abundance. The data shown is a representative FACS contour plot of four transplants per group, where CD19+ B220+ phenotype B cells were quantified through a live cell CD45+ gate.

Supplemental Figure 3

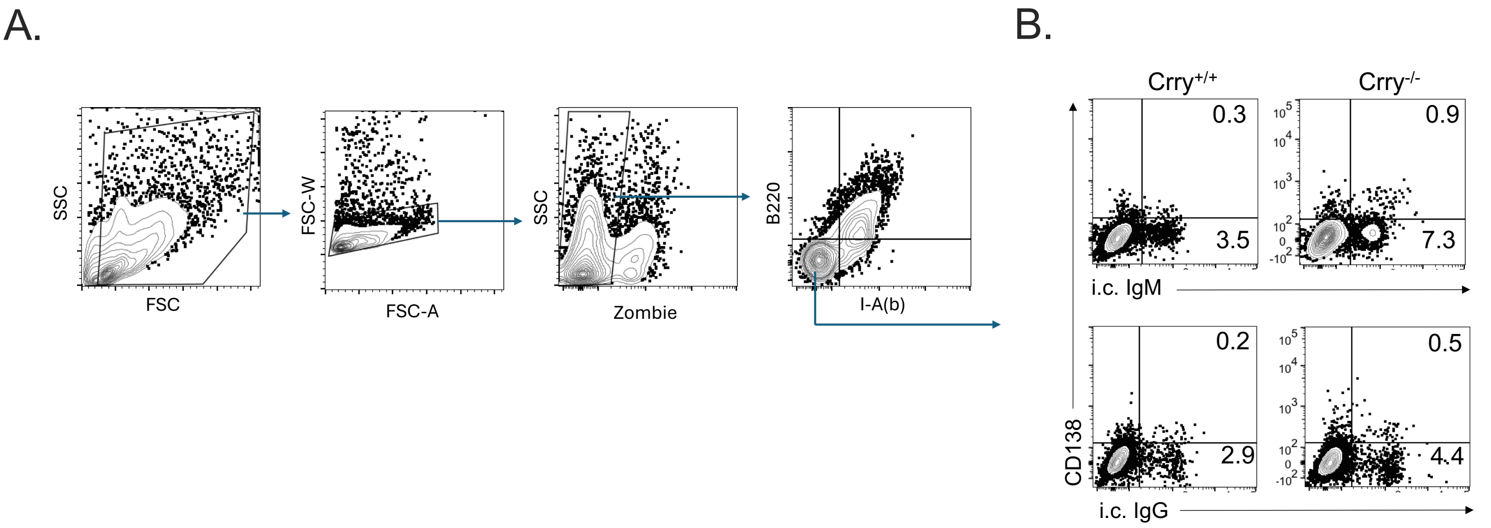

Supplemental Figure 3: Elevated antibody-secreting cell accumulation in allografts of Crry-deficient recipients. POD 16 lung allografts from wildtype and Crry-deficient recipients were assessed for the percent abundance of antibody-secreting cells. (A) FACS gating strategy to identify live I-A(b)$^{lo/-}$ B220$^{lo/-}$ intragraft cells that express (B) intracellular (i.c.) IgM and i.c. IgG CD138+ antibody-secreting cells. Results shown are representative FACS contour plots from five transplants per group.

Supplemental Figure 4

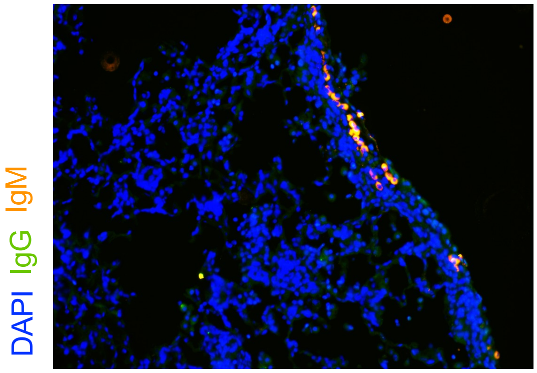

Supplemental Figure 4: IgM and IgG staining in the pleural cavity. POD 16 lung allograft from a Crry-deficient recipient with IgM+ and IgG+ cells within the pleural cavity. The image shown is a representative result from three lung transplants.

Supplemental Figure 5

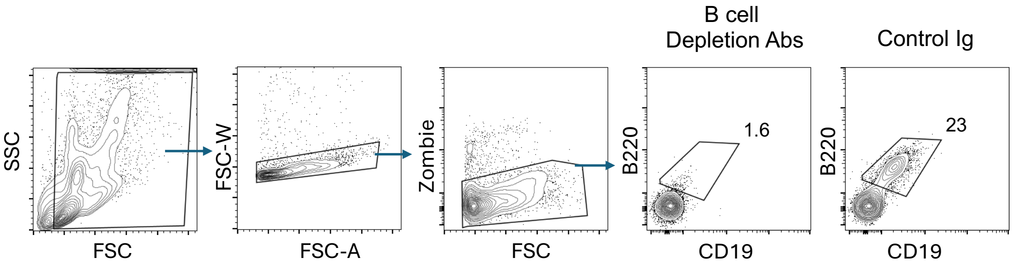

Supplemental Figure 5: B cell depletion of Crry-deficient lung allograft recipients. On POD 6 and 12, Crry-deficient lung recipients received a cocktail of B cell-depleting Abs or isotype control Abs and were evaluated for intragraft B cell abundance on a live CD19+ B220+ gate. The data shown are representative FACS contour plot results from 5 transplants per group.
