# Supplementary figures and images for: Impaired complement regulation drives chronic lung allograft dysfunction after lung transplantation
Source: J Clin Invest. 2025 Nov 11;136(1):e188891. doi: 10.1172/JCI188891 (PMC12721912; doi:10.1172/JCI188891)

Figure 3 (cropped)

A.

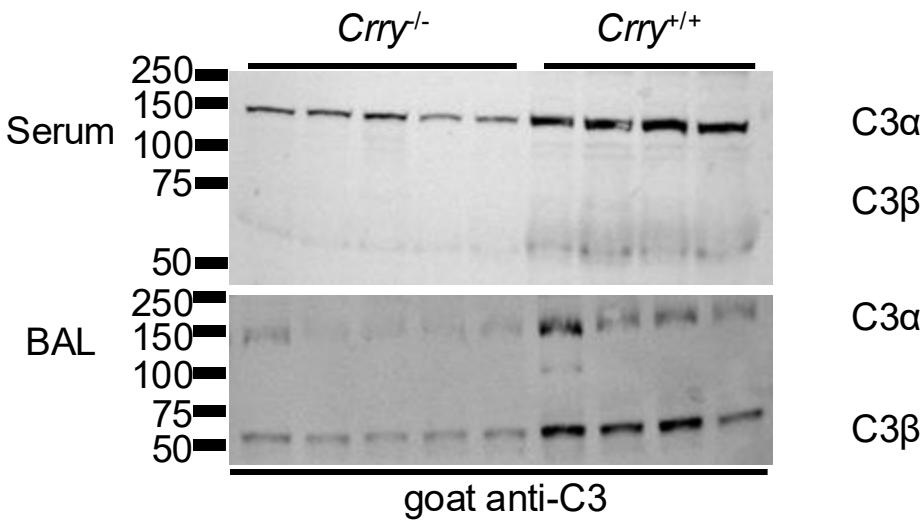

Figure 3

A.

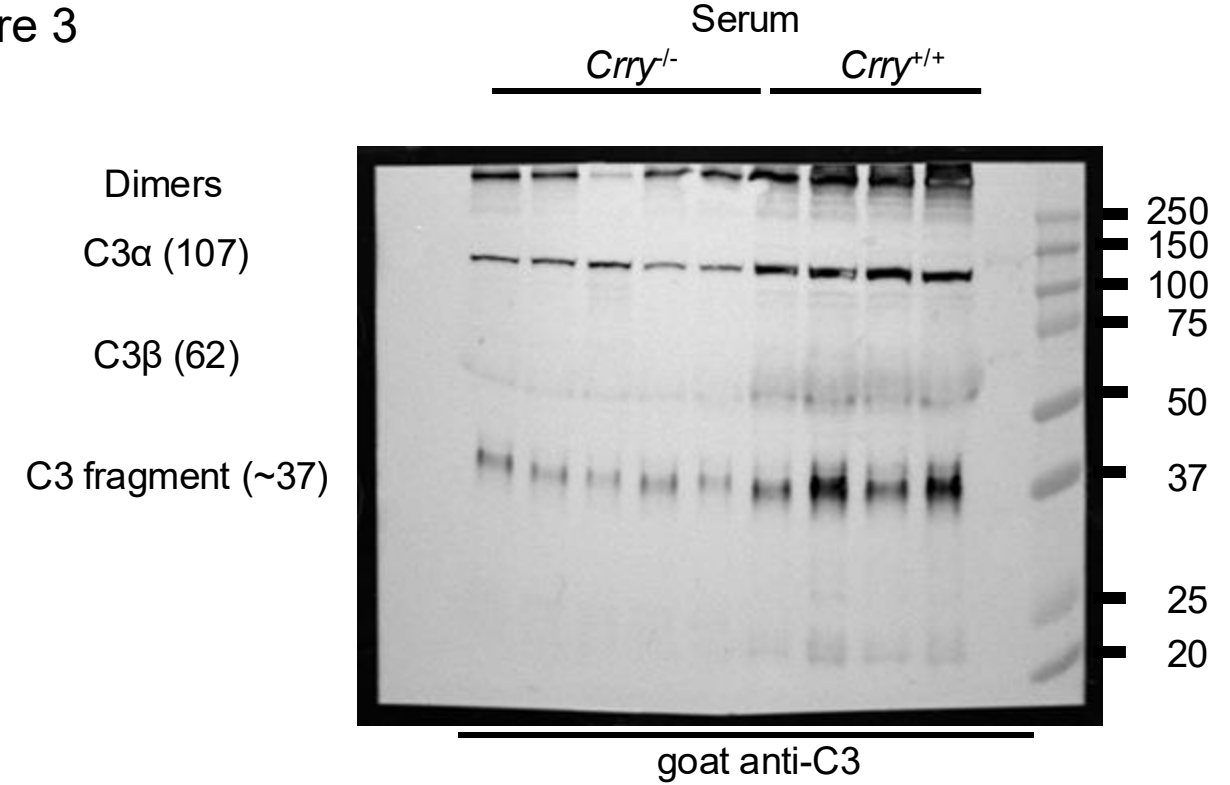

Figure 3

A.

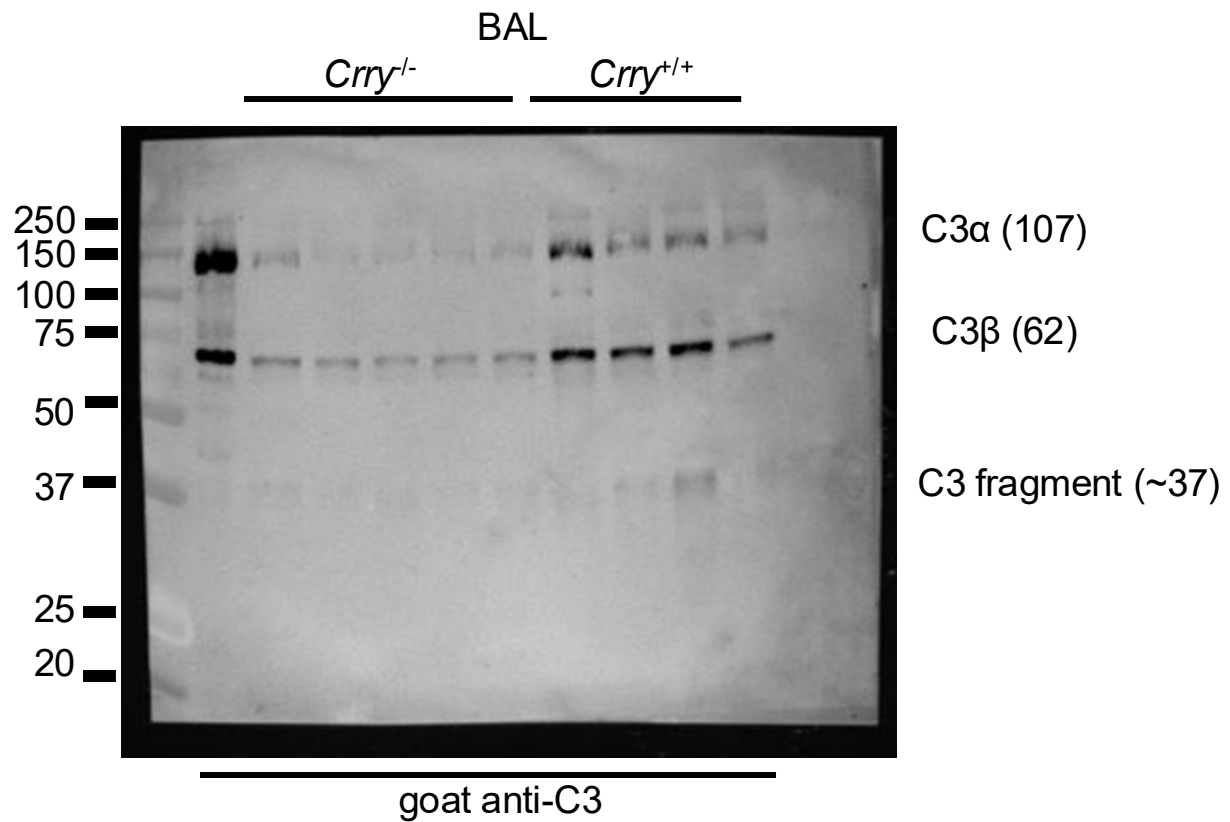

Supplement: Unedited blot and gel images [file jci-136-188891-s331.pdf]
